# Vagus Nerve Stimulation Improves Cardiac Function by Preventing Mitochondrial Dysfunction in Obese-Insulin Resistant Rats

**DOI:** 10.1038/srep19749

**Published:** 2016-02-01

**Authors:** Bencharunan Samniang, Krekwit Shinlapawittayatorn, Titikorn Chunchai, Wanpitak Pongkan, Sirinart Kumfu, Siriporn C. Chattipakorn, Bruce H. KenKnight, Nipon Chattipakorn

**Affiliations:** 1 Cardiac Electrophysiology Research and Training Center, Faculty of Medicine, Chiang Mai University, Chiang Mai, 50200, Thailand; 2 Cardiac Electrophysiology Unit, Department of Physiology, Faculty of Medicine, Chiang Mai University, Chiang Mai, 50200, Thailand; 3 Center of Excellence in Cardiac Electrophysiology, Faculty of Medicine, Chiang Mai University, Chiang Mai, 50200, Thailand; 4 Department of Oral Biology and Diagnostic Sciences, Faculty of Dentistry, Chiang Mai University, Chiang Mai, 50200, Thailand; 5 Emerging Therapies, Cyberonics Inc, Houston, Texas, USA

## Abstract

Long-term high-fat diet (HFD) consumption leads to not only obese-insulin resistance, but also impaired left ventricular (LV) function. Vagus nerve stimulation (VNS) has been shown to exert cardioprotection. However, its effects on the heart and metabolic parameters under obese-insulin resistant condition is not known. We determined the effects of VNS on metabolic parameters, heart rate variability (HRV) and LV function in obese-insulin resistant rats. Male Wistar rats were fed with HFD for 12 weeks, and were randomly divided into sham and VNS groups. VNS was applied for the next 12 weeks. Echocardiography, blood pressure and HRV were examined. Blood samples were collected for metabolic parameters. At the end, the heart was removed for determination of apoptosis, inflammation, oxidative stress, and cardiac mitochondrial function. VNS for 12 weeks significantly decreased plasma insulin, HOMA index, total cholesterol, triglyceride, LDL and visceral fat. Serum adiponectin was significantly increased in the VNS group. VNS also significantly decreased blood pressure, improved HRV and LV function, decreased cardiac MDA, TNF-α and Bax levels, and improved cardiac mitochondrial function. VNS improves metabolic and hemodynamic parameters, and the LV function via its ability against apoptosis, inflammation and oxidative stress, and preserved cardiac mitochondrial function in obese-insulin resistant rats.

Obesity rates have drastically increased across all age and socio-economic groups around the world^1^. Obesity is also an important factor that leads to the development of the metabolic syndrome which includes a constellation of risk factors for cardiovascular disease such as insulin resistance, dyslipidemia, hyperglycemia and hypertension^2^, and contributes to increased morbidity and mortality in the affected patients^3^. Growing evidence also demonstrates that increased inflammation and oxidative stress play a key role in metabolic syndrome^4^,^5^. Moreover, abnormal production and secretion of adipokines has been observed in metabolic syndrome^6^. It has been shown that all of these parameters can cause a decreased parasympathetic activity and increased risk of cardiovascular disease^7^.

Although pharmacological agents still play a vital role in both metabolic syndrome and cardiovascular disease treatments, they could cause serious adverse effects^8^. Growing evidence demonstrates that augmenting parasympathetic activity by vagus nerve stimulation (VNS) exerted a wide range of beneficial effects including cardioprotection and maintaining metabolic homeostasis^9^,^10^,^11^,^12^. Specifically, VNS has been shown to improve left ventricular (LV) function and prevent cardiac arrhythmia in a model of acute myocardial infarction through its anti-inflammatory and anti-apoptosis effects^11^,^12^,^13^,^14^. In addition to its cardioprotective effects, VNS has been shown to exert the beneficial effect in maintaining metabolic homeostasis by reducing the two-hour glucose tolerance in the impaired glucose tolerance patients^10^. Furthermore, chronic VNS has been shown to cause weight loss, as a result of its effect on eating behavior and appetite^15^, and reduced food intake, body weight and mass cells through an increase in vagal afferent satiety signals in obese rats^16^.

Although VNS treatment exerts cardioprotective and metabolic benefits, the effects of VNS on the heart in obese-insulin resistant condition have never been investigated. In the present study, we determined the effects of the long-term VNS treatment on the metabolic parameters and LV function in obese-insulin resistant rats. We tested the hypothesis that VNS improves insulin sensitivity and cardiac function by its ability to decrease oxidative stress, inflammation, apoptosis and cardiac mitochondrial dysfunction caused by obese-insulin resistance.

## Results

### Effects of obese-insulin resistance and VNS on blood pressure

**Figure 1:**
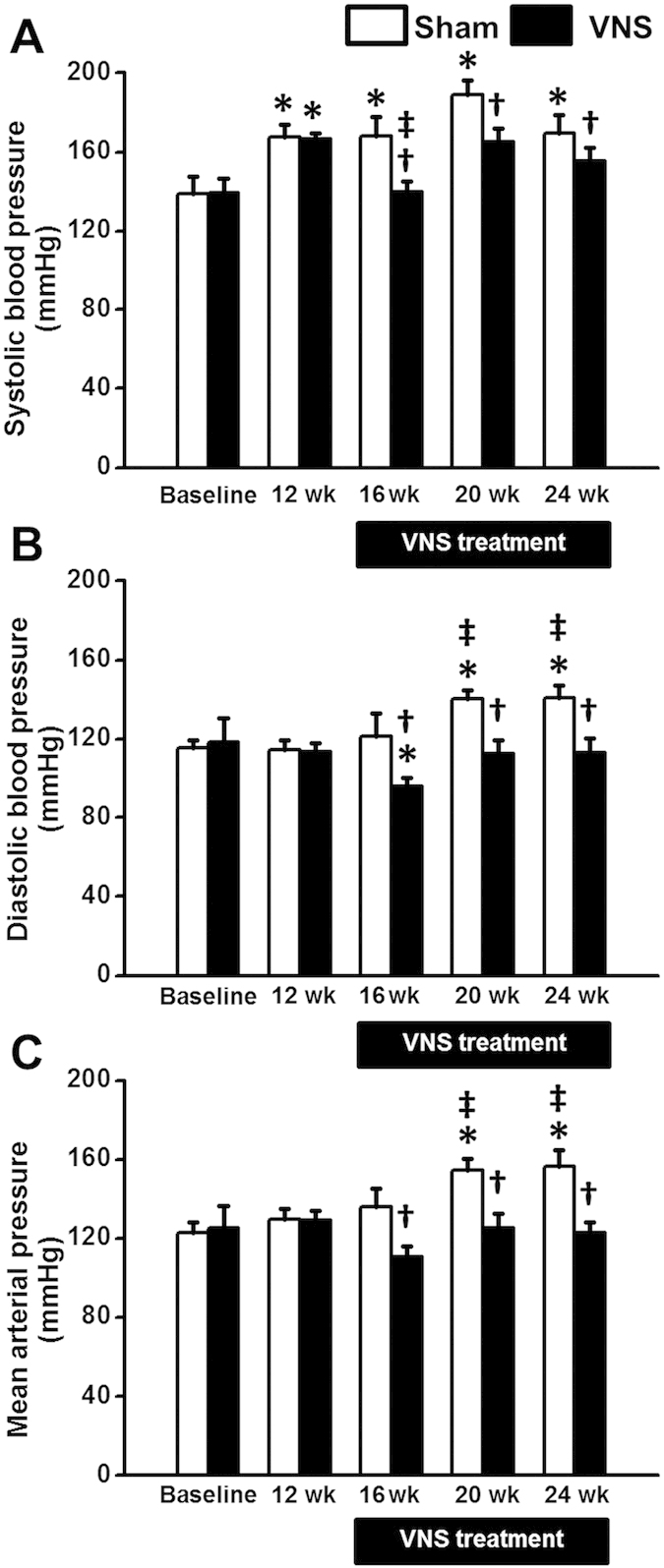
Effects of vagus nerve stimulation on blood pressure. (**A**) SBP increased significantly at week 12^th^ of HFD consumption. VNS significantly decreased SBP at week 16^th^, week 20^th^ and week 24^th^ in obese-insulin resistance. (**B**) DBP increased significantly at week 20^th^ of HFD consumption. VNS significantly decreased DBP at week 16^th^, week 20^th^ and week 24^th^ in obese-insulin resistance. (**C**) MAP increased significantly at week 20^th^ of HFD consumption. VNS significantly decreased MAP at week 16^th^, week 20^th^ and week 24^th^ in obese-insulin resistance. Data were expressed as mean ± SEM. *p < 0.05 vs baseline, †p < 0.05 vs sham group, ‡p < 0.05 vs at 12 week after high-fat feeding of the same group, n = 8/group.

At baseline, systolic blood pressure (SBP), diastolic blood pressure (DBP) and mean arterial pressure (MAP) were not different between sham and VNS groups (Fig. 1). SBP was significantly increased after 12 weeks of high-fat diet consumption, compared with baseline (Fig. 1A). DBP and MAP were significantly increased after 20 weeks of high-fat diet consumption (Fig. 1B,C). Interestingly, at 4 weeks after VNS treatment, there was a significant improvement of SBP, DBP and MAP in the VNS group (Fig. 1). Moreover, VNS significantly reduced SBP, DBP and MAP at weeks 4, 8 and 12 after VNS treatment (Fig. 1).

### Effects of obese-insulin resistance and VNS on cardiac function and HRV

**Figure 2:**
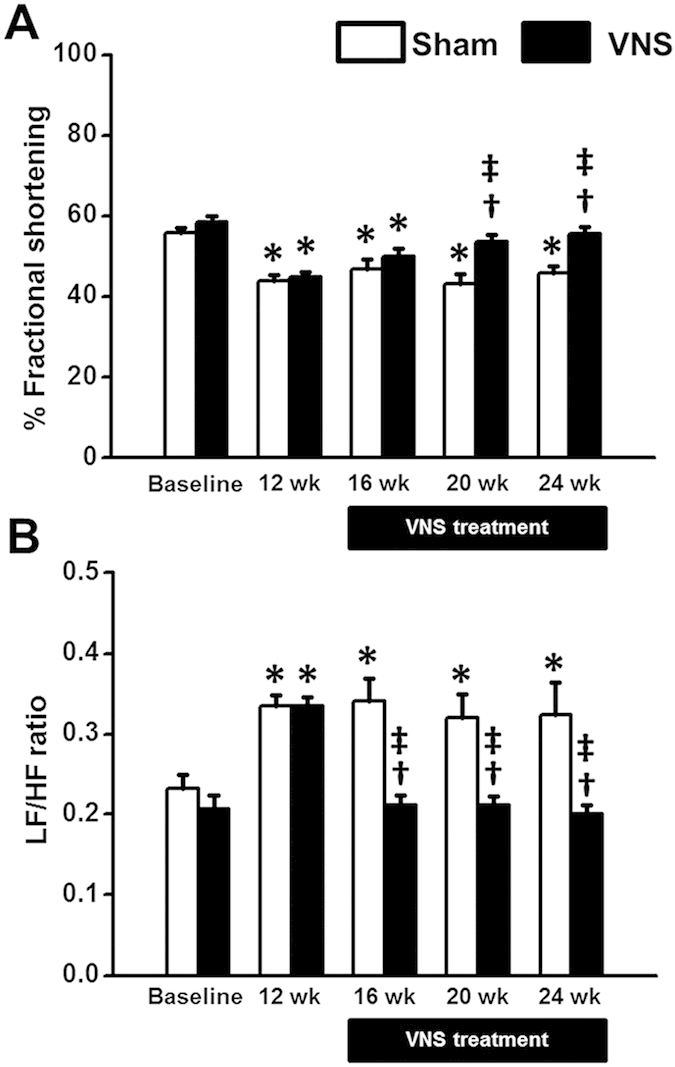
Effects of vagus nerve stimulation on left ventricular function. (**A**) %FS decreased significantly at week 12^th^ of HFD consumption. VNS significantly increased %FS at week 20^th^ and week 24^th^ in obese-insulin resistance. (**B**) LF/HF ratio increased significantly at week 12^th^ of HFD consumption. VNS significantly decreased LF/HF ratio at week 16^th^, week 20^th^ and week 24^th^ in obese-insulin resistance. Data were expressed as mean ± SEM. *p < 0.05 vs baseline, †p < 0.05 vs sham group, ‡p < 0.05 vs at 12 week after high-fat feeding of the same group, n = 8/group.

At baseline, %FS was not different between sham and VNS groups (Fig. 2A). After 12 weeks of high-fat diet consumption, %FS decreased significantly in both groups (Fig. 2A). In VNS group, %FS at weeks 8 and 12 after VNS treatment increased significantly (Fig. 2A). Although ESP and +dP/dt were not different among groups, VNS significantly reduced EDP and increased −dP/dt and SV, compared with the sham group (Table 1). Moreover, there was no difference in LV stroke work (SW) between the sham and the VNS groups.

For the HRV, the LF/HF ratio was not different between sham and VNS groups (Fig. 2B) at baseline. However, the LF/HF ratio significantly increased at week 12 after high-fat diet consumption (Fig. 2B), indicating cardiac sympathovagal imbalance. In the VNS group, the LF/HF ratio decreased significantly at weeks 4, 8 and 12 after VNS treatment (Fig. 2B), suggesting an improved cardiac sympathovagal balance by VNS.

### Effects of obese-insulin resistance and VNS on metabolic parameters

**Figure 3:**
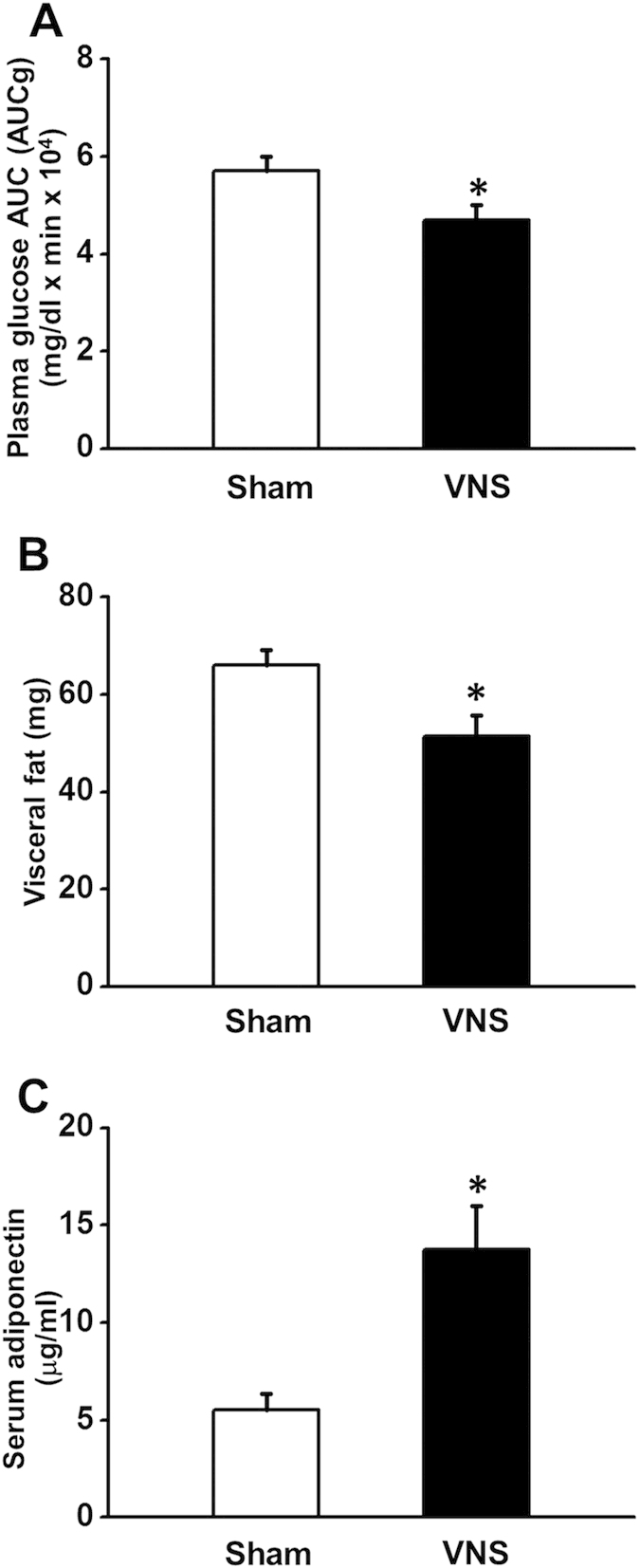
Effects of vagus nerve stimulation on plasma glucose, visceral fat and serum adiponectin after 24 weeks of HFD consumption. (**A**) VNS significantly decreased plasma glucose AUC in obese-insulin resistance. (**B**) VNS significantly decreased visceral fat in obese-insulin resistant. (**C**) VNS significantly increased serum adiponectin in obese-insulin resistance. Data were expressed as mean ± SEM. *p < 0.05 vs sham group, n = 8/group.

At baseline, metabolic parameters were not different between Sham and VNS groups (Table 2). After 12 weeks of high-fat diet consumption, the body weight, plasma insulin, HOMA index, plasma total cholesterol and plasma LDL levels significantly increased in both groups (Table 2). Plasma insulin, HOMA index, plasma total cholesterol, plasma triglyceride and plasma LDL levels decreased significantly after 12 weeks of VNS. No differences in body weight and food intake between the sham and VNS groups (Table 2). Moreover, after 12 weeks of VNS, plasma glucose AUC and visceral fat decreased significantly (Fig. 3), whereas the serum adiponectin level significantly increased in the VNS group (Fig. 3C).

### Effects of VNS on the oxidative stress, apoptosis and inflammation in obese-insulin resistance

**Figure 4:**
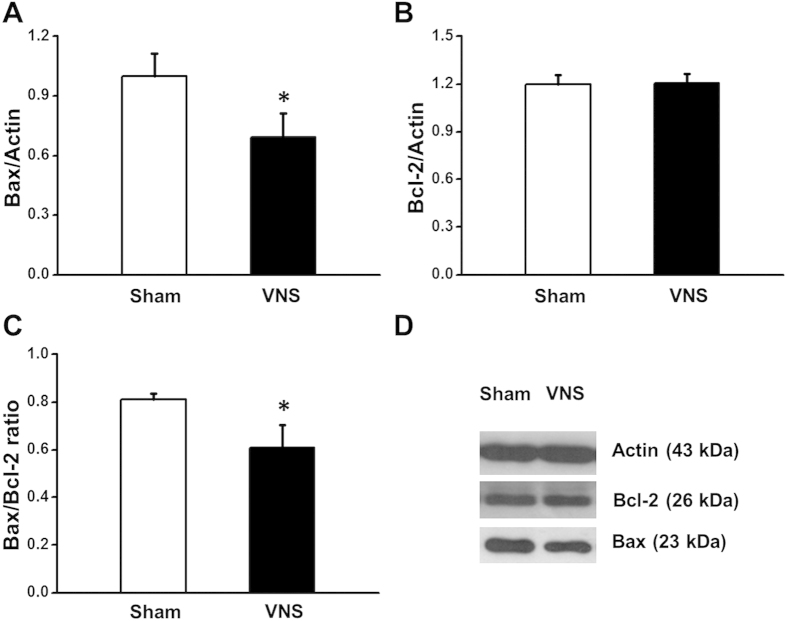
Effects of vagus nerve stimulation on Bax, and Bcl-2 expression after 24 weeks of HFD consumption. (**A**) VNS significantly decreased Bax expression in obese-insulin resistance. (**B**) VNS did not change the expression of Bcl-2 in obese-insulin resistance. (**C**) Bax/Bcl-2 ratio decreased significantly in VNS group in obese-insulin resistance. (**D**) Representative images showing band width of Bax, Bcl-2 and actin expression. Data were expressed as mean ± SEM. *p < 0.05 vs. sham group, n = 8/group. The full length blots are presented in supplementary Fig S1.

**Figure 5:**
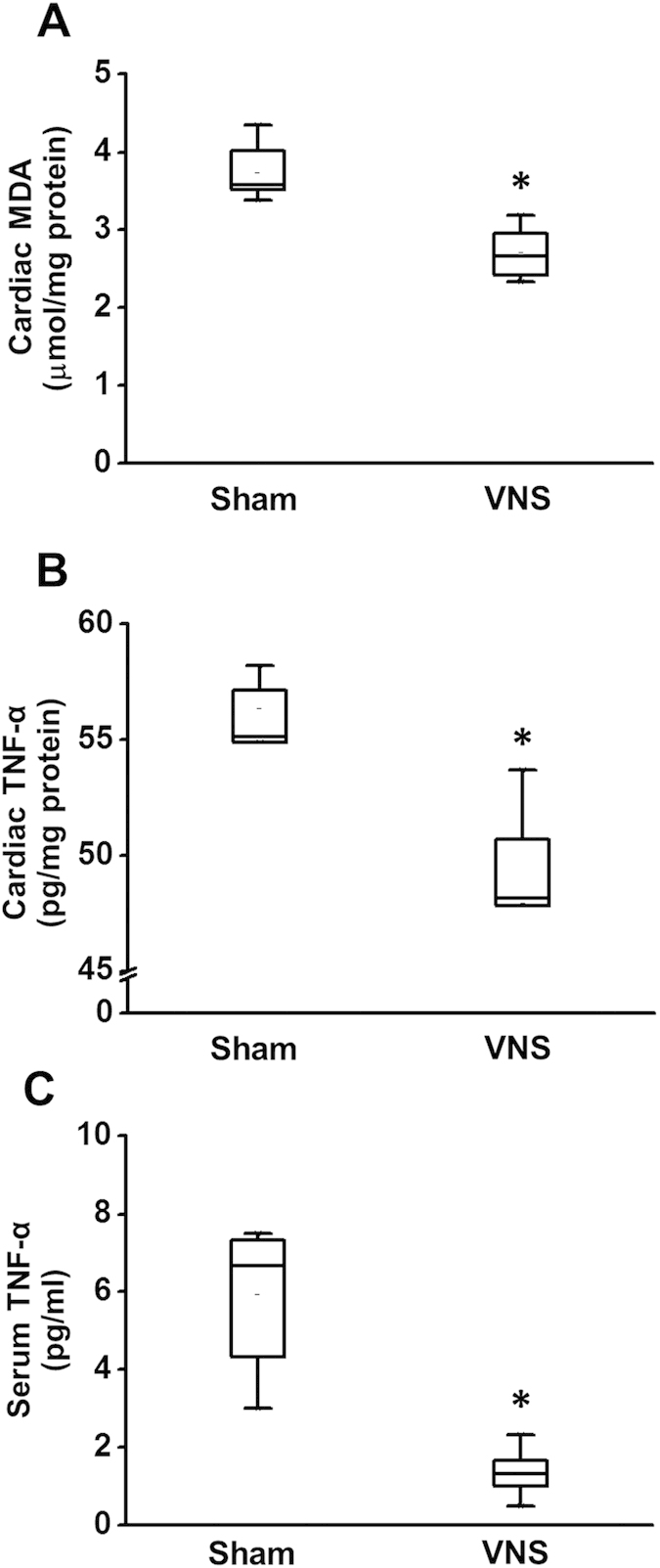
Box plots representing the effects of vagus nerve stimulation on cardiac MDA, cardiac TNF-α and serum TNF-α after 24 weeks of HFD consumption. (**A**) VNS significantly decreased cardiac MDA in obese-insulin resistance. (**B**) VNS significantly decreased cardiac TNF-α in obese-insulin resistance. (**C**) VNS significantly decreased serum TNF-α in obese-insulin resistance. *p < 0.05 vs. sham group, n = 8/group.

After 12 weeks of VNS treatment, VNS significantly decreased Bax expression compared with the sham group (Fig. 4A,D), whereas the Bcl-2 expression was not different among groups (Fig. 4B,D). However, the Bax/Bcl-2 ratio decreased significantly in the VNS group (Fig. 4C) suggesting that VNS attenuated cardiac apoptosis in obese-insulin resistance. Cardiac MDA level decreased significantly after 12 weeks of VNS treatment in the VNS group (Fig. 5A). Moreover, after 12 weeks of VNS treatment, VNS significantly reduced both cardiac and serum TNF-α levels compared with the sham group (Fig. 5B,C), suggesting that VNS attenuated cardiac inflammation and oxidative stress in obese-insulin resistance.

### Effects of VNS on cardiac mitochondrial function in obese-insulin resistance

**Figure 6:**
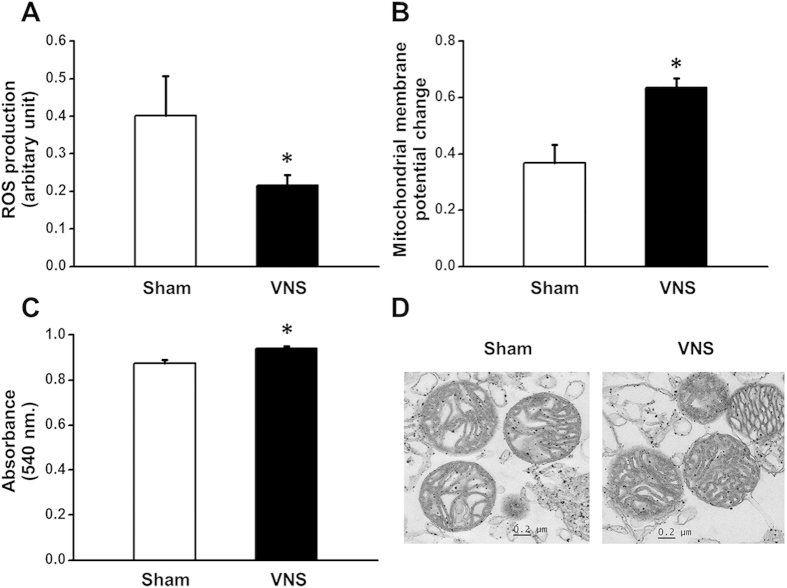
Effects of vagus nerve stimulation on cardiac mitochondrial function and morphology after 24 weeks of HFD consumption. (**A**) VNS significantly reduced mitochondrial ROS production in obese-insulin resistance. (**B**) VNS significantly increased mitochondrial membrane potential change in obese-insulin resistance. (**C**) VNS significantly increased cardiac mitochondrial absorbance in obese-insulin resistance. (**D**) Representative images showing VNS dramatically prevented mitochondrial swelling. Data were expressed as mean ± SEM. *p < 0.05 vs. sham group, n = 8/group.

After 12 weeks of VNS treatment, VNS significantly reduced mitochondrial ROS production compared with the sham group (Fig. 6A). For mitochondrial membrane potential change, VNS significantly increased the red/green fluorescent intensity ratio compared with the sham group (Fig. 6B), indicating a reduced mitochondrial depolarization. In addition, the absorbance increased significantly in the VNS group, suggesting an attenuated mitochondrial swelling (Fig. 6C). For cardiac mitochondrial ultrastructure, the unfolded cristae were mostly detected in the sham group (Fig. 6D, left). Interestingly, VNS effectively prevented mitochondrial swelling as shown by preserved cristae in obese-insulin resistance (Fig. 6D, right).

## Discussion

In this study, we investigated the effects of VNS on metabolic parameters and cardiac function in obese-insulin resistant animal model. The major findings of this study are as follows: 1) VNS effectively improved insulin resistant condition by increased insulin sensitivity, and increased serum adiponectin level, 2) VNS improved LV function and HRV impaired by obese-insulin resistant condition, 3) VNS significantly decreased blood pressure and 4) VNS exerted anti-oxidant, anti-apoptosis and anti-inflammation properties. Our results provide novel evidence that chronic VNS applied later after the presence obese-insulin resistant condition provides effective cardioprotection and improved metabolic parameters in an obese-insulin resistant model.

In the present study, rats developed hypertension and insulin resistance after 12 weeks of HFD consumption. This finding is consistent with our previous studies in which long-term HFD consumption induces insulin resistance, as well as increases oxidative stress and induces cardiac mitochondrial dysfunction^17^,^18^,^19^. A previous study has shown the presence of an autonomic imbalance shifting toward augmented sympathetic tone during the development from normal glucose tolerance to impaired glucose tolerance and finally diabetes^20^. Metabolic syndrome also has been shown to have an inverse correlation with the vagal component of high-frequency component of the HRV^21^. Thus, rebalancing the autonomic system by VNS may lead to the attenuation of the metabolic parameters in our study. Moreover, in the present study, VNS also significantly increased serum adiponectin and decreased visceral fat after 12 weeks of VNS treatment. Adiponectin is a bioactive adipokine that is expressed in adipose tissues and directly sensitizes the body to plasma insulin. In addition, adiponectin also regulates the energy homeostasis, glucose and lipid metabolism^22^. Previous studies have shown that plasma adiponectin level is lower in patients with diabetes, hypertension, and dyslipidemia^23^,^24^,^25^. Accumulating evidence indicates that adiponectin directly interacts with cardiovascular tissues and prevents cardiovascular pathology. Therefore, the beneficial effects of VNS on metabolic and hemodynamic parameters might be due to its ability to increase adiponectin in obese-insulin resistant condition.

Previous study has shown that central obesity causes an increase in sympathetic activity^26^. Furthermore, when patients have both metabolic syndrome and obesity, the level of sympathetic activity is even higher^27^. In the present study, we found that the insulin-resistant condition and obesity were developed at week 12 after HFD consumption. In addition, the LF/HF ratio increased significantly at week 12 following HFD consumption, indicating cardiac sympathovagal disturbance. Increased oxidative stress has been shown to strongly influence cardiac autonomic regulation^28^. The increased oxidative stress in our obese-insulin resistant rats could contribute to the impaired cardiac autonomic balance observed in this study.

A growing number of studies have also shown the strong correlation between metabolic syndrome and ROS production^29^. In a rat model, HFD induced obesity significantly increases the blood pressure and oxidative stress level^5^. Moreover, an increased ROS production can induce mitochondrial dysfunction, apoptosis and cell death^30^. Cardiac mitochondria supply energy to produce the proper cardiac contraction and relaxation on a beat to beat basis. Previous study has shown that worsening of intrinsic myocardial contraction in the transition from obesity to diabetes mellitus is likely related to cardiac mitochondrial dysfunction^31^. Moreover, our previous studies have shown that long-term HFD consumption can cause LV dysfunction due to mitochondrial dysfunction, oxidative stress and inflammation^17^,^19^. Moreover, TNF-α increased significantly during the development of obesity and metabolic syndrome^4^,^32^.

In the present study, we found that VNS significantly reduced cardiac and serum TNF-α levels after VNS application for 12 weeks. Previous study has shown that VNS inhibits the synthesis of TNF-α and decreases inflammatory responses via nicotinic receptor activation leading to the inhibition of other inflammatory cytokines release^33^. Furthermore, the cardiac MDA level also decreased significantly in the VNS group in our study. Moreover, VNS significantly improved cardiac mitochondrial function impaired by obese-insulin resistance. This beneficial effect of VNS could be exerted through inhibition of opening of mitochondrial permeability transition pore as shown in the previous study^34^. The results obtained from this study indicate the cardio-metabolic protective benefits of VNS in the obese-insulin resistant rats induced by long-term high-fat diet consumption. Moreover, its contribution on cardiac mitochondrial protection could be an important underlying mechanism of this protection on the heart. In addition, the influence of vascular bed alterations in obesity may also contribute to these effects. Since VNS improved cardiac mitochondrial function, decreased oxidative stress, reduced apoptosis and inflammation, this could be the responsible mechanisms for an improved LV function in obese-insulin resistant rats observed in the present study.

Long-term HFD consumption obviously induced obese-insulin resistance, LV dysfunction, increased oxidative stress, inflammation, and cardiac autonomic imbalance. Under obese-insulin resistance condition, chronic VNS improved LV contractile function, cardiac autonomic regulation and metabolic parameters via its ability against apoptosis, oxidative stress, inflammation, cardiac mitochondrial dysfunction, and increased serum adiponectin level. Moreover, VNS significantly reduced blood pressure by increase parasympathetic activity in obese-insulin resistant rats. These findings suggest that VNS may be beneficial as a novel adjuvant therapeutic approach in obese-insulin resistant patients.

## Methods

### Animals and diet

All experiments in this study were approved by the Faculty of Medicine, Chiang Mai University Institutional Animal Care and Use Committee, in compliance with NIH guidelines, and in accordance with the ARRIVE guidelines for reporting experiments involving animals^35^. In this study, male Wistar rats (180–200 g) were obtained from the National animal center, Salaya campus, Mahidol University, Bangkok, Thailand. Rats were fed with high-fat diet for 12 weeks then rats were randomly divided into 2 groups, sham group (n = 8) and VNS group (n = 8). After 12-week of long-term high-fat diet consumption, all rats (500–580 g) were subjected to VNS implantation. Rats were fully recovered at 3–5 days after surgery. After 1 week of recovery period, VNS will be stimulated for 12 weeks. At week 12^th^ of high-fat diet feeding and weeks 4^th^, 8^th^, 12^th^ of VNS treatment, blood samplings were collected for the investigations of glucose, total cholesterol, triglyceride, low density lipoprotein (LDL), high density lipoprotein (HDL), and insulin levels. Heart rate variability (HRV), blood pressure (BP) and echocardiography were also examined. At the end of the study, pressure-volume (P-V) loops were analyzed. Then, rats were sacrificed by decapitation and the heart were rapidly removed and homogenized for mitochondrial and biochemical studies including western blotting for Bax, Bcl-2, and actin.

### Vagus nerve stimulation

Rats were anesthetized with xylazine (3 mg/kg) and zoletil (50 mg/kg). After hair shaving and skin cleaning, a bipolar cuff electrode were implanted around the left cervical vagus nerve and connected to an implantable pulse generator (Demipulse, Model 103, Cyberonics). A period of one week was allowed for recovery from the surgical implantation of the stimulation system. VNS was continuously delivered at 20 Hz, 500 μs pulse width, 0.5–0.75 mA, turn ON 14 s and turn OFF 48 s (23% duty cycle). These VNS parameters were sufficient to produce a 5% reduction in heart rate, and the effect of VNS was completely disappeared within 20 seconds after the cessation of VNS as evidenced by the decreased heart rate back to the pre-stimulation values. Prior to data collection, VNS was turned off for 5–10 min and remained off for duration of the hemodynamic evaluations, echocardiographic, BP and HRV measurements. In the sham group, similar surgical procedure were done except that the programmed VNS was turn off.

### Measurement of blood pressure (BP)

Rats were preheated under infrared warming pad for at least 5 minutes to dilate the tail vein and acclimate to the holder. Blood pressure measurement was performed by using a CODA2 channel non-invasive blood pressure system (Kent Scientific Corporation, Wyoming, USA). A blood pressure value was achieved from taking an average of 20 consecutive measurements at a steady state^36^. Blood pressure was measured at baseline, 12, 16, 20 and 24 weeks.

### Echocardiography

Animals were lightly anesthetized and placed in a supine position. An echocardiography probe (S12, Hewlett Packard) which was connected to an echocardiography machine (SONOS 4500, Philips) was gently placed on the chest and moved for collecting the data along short axis of the heart. Signals from M-mode echocardiography at the level of papillary muscles were recorded. Then, the percentage of the fractional shortening (%FS) was calculated^17^. Echocardiogram was recorded at baseline, 12, 16, 20 and 24 weeks.

### Heart rate variability (HRV)

Lead II Electrocardiograms (ECG) was recorded for 20 minutes in conscious animals using a signal transducer (PowerLab 4/25T, ADInstrument) and operated through Chart 5.0 program. ECG data was then analyzed using the frequency-domain method by the MATLAB program. High-frequency (HF) component was considered as a marker of parasympathetic tone while low-frequency (LF) component was considered as a marker of parasympathetic tone and sympathetic tone. The cardiac sympathetic/parasympathetic balance was reported as the LF/HF ratio. Increased LF/HF ratio indicates cardiac sympathovagal imbalance^37^,^38^. HRV was recorded at week 0 (baseline), 12, 16, 20 and 24.

### Determination of metabolic parameters

Plasma insulin level was measured using a commercial sandwich ELISA kit (Millipore, MI, USA)^18^,^39^. Insulin resistance was assessed by Homeostasis Model Assessment (HOMA) which calculated from fasting plasma insulin and fasting plasma glucose concentration. Higher HOMA index shows a higher degree of insulin resistance^39^. Plasma glucose, total cholesterol, and triglyceride levels were determined using a commercial colorimetric assay kit (Biotech, Bangkok, Thailand)^18^,^39^. Plasma HDL level was determined using a commercial colorimetric assay kit (Biovision, California, USA)^17^, and plasma LDL level was calculated using Friedewald equation^40^. Serum adiponectin level was determined using a commercial sandwich ELISA kit (Invitrogen, Frederick, USA)^41^.

### Left ventricular pressure-volume loops (P-V loops) analysis

At the end of the study, rats were anesthetized by intramuscular injection of the combination of zoletil (50 mg/kg) and xylazine (3 mg/kg)^42^. LV function was evaluated using a pressure-volume (P-V) conductance catheter (1.9F; Scisense Instrument, Ontario, Canada). The catheter was inserted into the right carotid artery and advanced into the LV chamber to record LV pressure and volume relationship. Heart rate (HR), left ventricular end-systolic pressure (LVESP), left ventricular end-diastolic pressure (LVEDP), maximal slope of the systolic pressure increment (+dP/dt), maximal slope of the diastolic pressure decrement (−dP/dt), stroke volume (SV) and stroke work (SW) were assessed^42^,^43^.

### Determination of cardiac malondialdehyde (MDA) level

Malondialdehyde (MDA) concentration in cardiac tissue was measured by a HPLC system (Thermo scientific, Bangkok, Thailand)^44^. A sample was mixed with H_3_PO_4_ and thiobabituric acid (TBA) solution to generate thiobarbituric acid reactive substances (TBARS). Cardiac TBARS concentration was determined directly from standard curve and reported as MDA equivalent concentration^45^.

### Determination of cardiac mitochondrial function

At the end of the study, rats were deeply anesthesized by intramuscular injection of the combination of zoletil (50 mg/kg) and xylazine (3 mg/kg). Then, the heart was rapidly removed, and chopped into small pieces on iced-cold plate. Cardiac tissues were homogenized in ice-cold isolated heart buffer which contained (in mmol/l) sucrose 300, TES 5 and EGTA 0.2, pH 7.2 (4 °C). The homogenate was centrifuged at 800 *g* for 5 minutes, and the supernatant was collected and then centrifuged at 8,800 *g* for 5 minutes. The pellet was resuspended in ice-cold buffer and centrifuged at 8,800 *g* for 5 minutes. Isolated mitochondria were collected and the protein concentration were determined^46^.

#### Cardiac mitochondrial ROS production

The production of reactive oxygen species (ROS) in cardiac mitochondria were measured by dichlorohydrofluorescein diacetate (DCFDA) dye staining. The mitochondria were incubated with 2 μM DCFDA for 25 minutes in 25 °C. The ROS level was assessed at the wavelength of 485 nm (bandwidth 5 nm) and emission wavelength at 530 nm (bandwidth 10 nm) using a fluorescent microplate reader (BioTek Instruments, Winooski, Vermont, USA). Increased fluorescent intensity indicates increased mitochondrial ROS production^46^.

#### Cardiac mitochondrial membrane potential changes

Mitochondria membrane potential were measured by staining with the dye 5,5′,6,6′ tetrachloro-1,1′,3,3′-tetraethylbenzimidazolcarbocyanine iodide (JC-1). Isolated cardiac mitochondria were stained with JC-1 (5 μM) at 37 °C for 30 minutes, and then the membrane potential was determined as fluorescence intensity by using a fluorescent microplate reader. The change in mitochondrial membrane potential was calculated as the ratio of red to green fluorescence. Decreased red/green fluorescent ratio indicates mitochondrial depolarization^46^.

#### Cardiac mitochondrial swelling

Isolated cardiac mitochondria were incubated in 1.5 ml of respiration buffer (constaining 100 mM KCl, 50 mM sucrose, 10 mM HEPES, 5 mM KH_2_PO_4_, pH 7.4 at 37 °C) with addition of 10 mM pyruvate/malate for 1 minute. Mitochondrial swelling were evaluated by measuring the change of absorbance, using spectrophotometer, for 30 minutes and detected by decreasing of absorbance of suspension at 540 nm^46^.

#### Cardiac mitochondria morphology

Isolated cardiac mitochondria were fixed with 2.5% glutaraldehyde in a 0.1-M phosphate buffer overnight and post fixed in a 1% cacodylate-buffer osmium tetroxide, then dehydrated with graded series of ethanol. Cardiac mitochondria morphology was determined using a transmission electron microscope^46^.

### Western blot analysis

Myocardial tissues were homogenized in a lysis buffer (containing 1% Nonidet P-40, 0.5% sodium deoxycholate, 0.1% sodium dodecyl sulfate (SDS) in 1xPBS) in order to extract protein. Total protein were mixed with the loading buffer (consist of 5% mercaptoethanol, 0.05% bromophenol blue, 75 nM Tris-HCl, 2% SDS and 10% glycerol with pH 6.8) in 1 mg/ml proportion. The mixture were boiled for 5 minutes and loaded into 10% gradient SDS-polyacrylamide gel. After that, proteins were transfer to a polyvinyldene difluoride (PVDF) membrane with the presence of glycine/methanol transfer buffer (containing 20 mM Tris base, 0.15 M glycine and 20% methanol) in a transfer system (Bio-Rad). PVDF membranes were incubated in 5% skim milk in 1xTBS-T buffer (containing 20 mM Tris-HCl (pH 7.6), 137 nM NaCl, 0.05% Tween-20) for 1 hour at room temperature^43^. The membrane were exposed to anti-Bax, anti-Bcl-2 (Cell Signaling Technology, Danvers, MA, USA) and anti-actin (Sigma-Aldrich, St. Louis, MO, USA) for 12 hours. Bound antibody was detected by horseradish peroxidase conjugated with anti-mouse IgG. Enhanced chemiluminescence (ECL) detection reagents were administrated to visualize peroxidase reaction products^42^.

### Inflammation analysis

TNF-α concentration in cardiac tissues and serum were measured by using an ELISA kit (Invitrogen, CA, USA). During the first incubation, the Rat TNF-α antigen binds to the immobilized antibody on one site. During the second incubation, this antibody binds to the immobilized Rat TNF-α captured during the first incubation. The intensity of this colored product is directly proportional to the concentration of Rat TNF-α present in the original specimen^47^.

### Statistical Analysis

Data were expressed as mean ± SEM. One-way ANOVA followed by Fisher’s least significant difference post-hoc was used to test the different among groups. Mitochondrial function was analyzed using Kruskal-Wallis H followed by Mann-Whitney U test. *P* < 0.05 was considered statistical significant.

## Additional Information

**How to cite this article**: Samniang, B. *et al.* Vagus Nerve Stimulation Improves Cardiac Function by Preventing Mitochondrial Dysfunction in Obese-Insulin Resistant Rats. *Sci. Rep.* **6**, 19749; doi: 10.1038/srep19749 (2016).

## Supplementary Material

Supplementary Information

## Figures and Tables

**Table 1:** Effects of vagus nerve stimulation on cardiac function after 24 weeks of HFD consumption.

Cardiac function	Sham	VNS
Heart rate (bpm)	302.1 ± 26.4	279.9 ± 10.1
ESP (mmHg)	133.2 ± 4.6	103.6 ± 11.3
EDP (mmHg)	24.3 ± 3.5	8.4 ± 1.9*
+dP/dt (mmHg/s)	11846.9 ± 2130.81	9026.6 ± 1205.4
−dP/dt (mmHg/s)	−12129.3 ± 2638.9	−29903.1 ± 5629.3*
SV (μl)	486.1 ± 18	619.8 ± 15*
SW (mmHg.μl)	57626.2 ± 1462.4	53635.9 ± 2847.9

Data were expressed as mean ± SEM. *p < 0.05 vs. sham group, n = 8/group.

ESP, end-systolic pressure; EDP, end-diastolic pressure; +dP/dt, maximal slope of the systolic pressure increment; −dP/dt, maximal slope of the diastolic pressure decrement; SV, stroke volume and SW, stroke work.

**Table 2:** Effects of vagus nerve stimulation on metabolic parameters.

Metabolic parameters	Baseline	After HF 12 weeks	Post VNS 12 weeks	
			Sham	VNS
Body weight (g)	261.1 ± 1.6	544.5 ± 6*	612.9 ± 9.6*	599.1 ± 10.6*
Food intake (g/day)	27.3 ± 0.5	20.8 ± 0.5*	20.4 ± 1.8*	20.2 ± 1.3*
Plasma glucose (mg/dl)	128.7 ± 6.7	127.9 ± 5.8	133.6 ± 4.3	133.7 ± 8.8
Plasma insulin (ng/ml)	1.7 ± 0.2	5.3 ± 0.3*	4.9 ± 1.4*	2.7 ± 0.2*^,^^†^^,^^‡^
HOMA index	9.2 ± 1.1	33.4 ± 5.5*	30.9 ± 10.8*	17.4 ± 2.9*^,^^†^^,^^‡^
Plasma total cholesterol (mg/dl)	61.8 ± 2	76.3 ± 2.7*	107.6 ± 7.5*^,†^	79.8 ± 2.7*^,‡^
Plasma triglyceride (mg/dl)	32.1 ± 2.1	30.6 ± 2.1	30.2 ± 3.3	21.4 ± 1.6*^,^^†^^,^^‡^
Plasma HDL (mg/dl)	23.5 ± 1.6	24.7 ± 1	25.3 ± 2.1	28.6 ± 1
Plasma LDL (mg/dl)	32.5 ± 2.2	46.1 ± 2.1*	66.4 ± 6*^,†^	48.6 ± 1.6*^,‡^

Data were expressed as mean ± SEM. *p < 0.05 vs. baseline, ^†^p < 0.05 vs. after HF 12 weeks, ^‡^p < 0.05 vs. sham group, n = 8/group.
