# Supplementary material for: Vagus Nerve Stimulation Improves Cardiac Function by Preventing Mitochondrial Dysfunction in Obese-Insulin Resistant Rats
Source: Sci Rep. 2016 Feb 1;6:19749. doi: 10.1038/srep19749 (PMC4735283; doi:10.1038/srep19749)
Supplement: Supplementary Information [file srep19749-s1.pdf]

Supplementary Information file

Title: Vagus Nerve Stimulation Improves Cardiac Function by Preventing Mitochondrial Dysfunction in Obese-Insulin Resistant Rats

Authors: Bencharunan Samniang, Krekwit Shinlapawittayatorn, Titikorn Chunchai, Wanpitak Pongkan, Sirinart Kumfu, Siriporn C. Chattipakorn, Bruce H. KenKnight PhD, Nipon Chattipakorn*

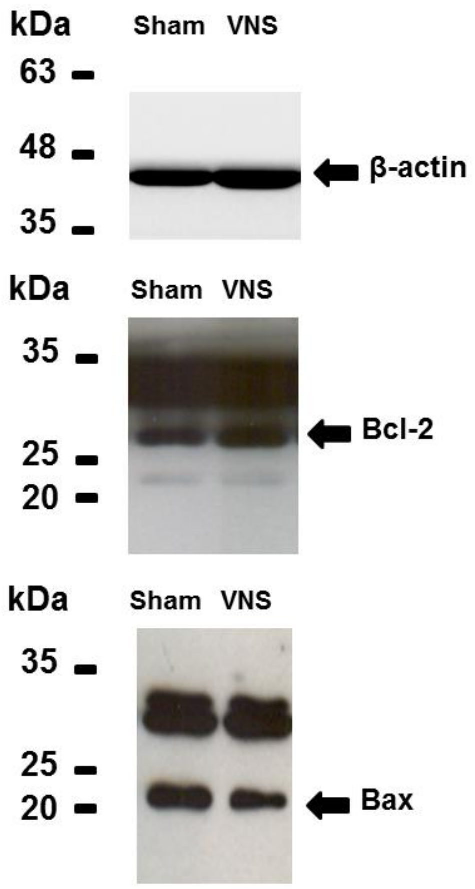

Supplementary Figure S1. Full length blots of data shown in Fig. 4D

The gels were initially cut ranged from 17 kDa to 63 kDa. *VNS* vagus nerve stimulation.
